# Exploring sex differences in drug use, health and service use characteristics among young urban crack users in Brazil

**DOI:** 10.1186/s12939-014-0070-x

**Published:** 2014-08-28

**Authors:** Neilane Bertoni, Chantal Burnett, Marcelo Santos Cruz, Tarcisio Andrade, Francisco I Bastos, Erotildes Leal, Benedikt Fischer

**Affiliations:** Institute of Psychiatry, Federal University of Rio de Janeiro, Rio de Janeiro, Brazil; Department of Community and Family Health, Federal University of Bahia, Salvador, Brazil; Institute of Communication and Scientific Information & Technology for Health, Oswaldo Cruz Foundation, Rio de Janeiro, Brazil; Centre for Applied Research in Mental Health and Addiction, Faculty of Health Sciences, Simon Fraser University, Vancouver, Canada; Social & Epidemiological Research, Centre for Addiction and Mental Health, Toronto, Canada

**Keywords:** Crack use, Gender, Health, Interventions, Brazil, Marginalized populations

## Abstract

**Introduction:**

Studies have shown important gender differences among drug (including crack) users related to: drug use patterns; health risks and consequences; criminal involvement; and service needs/use. Crack use is prevalent in Brazil; however, few comparative data by sex exist. We examined and compared by sex key drug use, health, socio-economic indicators and service use in a bi-city sample of young (18–24 years), regular and marginalized crack users in Brazil.

**Methods:**

Study participants (total n = 159; n = 124 males and n = 35 females) were recruited by community-based methods from impoverished neighborhoods in Rio de Janeiro and Salvador. Assessments occurred by an anonymous interviewer-administered questionnaire and serum collection for blood-borne virus testing between November 2010 and June 2011. Descriptive statistics and differences for key variables by sex were computed; in addition, a ‘chi-squared automatic interaction detector’ (‘CHAID’) analysis explored potential primary factors differentiating male and female participants.

**Results:**

Most participants were non-white, and had low education and multiple income sources. More women had unstable housing and income from sex work and/or panhandling/begging, whereas more men were employed. Both groups indicated multi-year histories of and frequent daily crack use, but virtually no drug injection histories. Men reported more co-use of other drugs. More women were: involved in sex-for-drug exchanges; Blood-Borne Virus (BBV) tested and HIV+. Both groups reported similar physical and mental health patterns; however women more commonly utilized social or health services. The CHAID analysis identified sex work; paid work; begging/panhandling; as well as physical and mental health status (all at p < 0.05) as primary differentiating factors by sex.

**Conclusions:**

Crack users in our study showed notable differences by sex, including socio-economic indicators, drug co-use patterns, sex risks/work, BBV testing and status, and service utilization. Results emphasize the need for targeted special interventions and services for males and female crack users in Brazil.

## Introduction

Research has documented a number of important differences between male and female users of – both licit and illicit – substances. While for many substance categories, women typically feature lower prevalence levels of use and problems than men, there appear to be some important differences in pathways to problems [1,2]. For example, several key studies document that women more quickly progress from use onset to problematic use (‘telescoping’), including manifested substance use disorders or need for treatment [3–5].

Women develop more severe medical or health problem consequences from substance use than men [6,7]. While women are typically less involved in substance use-related overt deviance (e.g., violence or crime), data from several studies suggest higher levels of co-morbid psychological or psychiatric problems among women users; specifically, large (30%–50%) proportions of women substance users report a history of (sexual or other) trauma and/or related Post-traumatic stress disorder (PTSD) [1,2,8]. In addition, larger proportions of women indicate co-morbid mood (e.g., depression) and/or anxiety disorders; however, the evidence is inconsistent about the directionality or sequence of these co-morbidities [1,9–11].

Data suggests that biology-related sex differences may influence differential drug use or problem patterns; for example, women may be more vulnerable to the reinforcing effects of psycho-stimulants during key developmental phases leading to dependence [12]. Women appear to enter substance use treatment less frequently than men, but commonly enter treatment driven by different factors (e.g., related to child care concerns), implying differential treatment needs. While a lack of targeted services for women drug users has been described for several countries, gender does not seem to majorly affect treatment retention, completion or outcomes [5,13–17].

Crack users are a specific population of substance users for which distinct gender differences have been reported. Many female users report initiation into crack use by an intimate partner, and use patterns and risks are commonly shaped by the power dynamics within an intimate relationship with a drug-using partner [18,19]. Women crack users commonly report more frequent or problematic (e.g., ‘bingeing’) crack use [20–22] as well as substantially more pronounced sexual risk behaviors related to crack use; for example, they are more commonly involved in sex work, and/or risky sexual behaviors (e.g., unprotected sex) in exchange for money or crack [23–28]. These gendered risk behavior patterns have translated into a higher prevalence of sex-risk related health problems – including higher prevalence of Blood-Borne Viruses (BBVs) or Sexually Transmitted Diseases (STDs) – among female crack user populations (e.g., Human Immunodeficiency Virus (HIV) and/or Hepatitis C Virus (HCV)) [22,29,30]. Overall, female crack users commonly feature lower self-reported health status than their male counterparts [25,31]; however there do not seem to be major gender-differences in treatment seeking, retention or outcomes [13,32].

Crack use has become a prevalent street drug use phenomenon in Brazil in recent years, associated with considerable health and social harms, as well as intensive discussions about appropriate interventions [33–36]. While previous estimates have been higher, a recent study estimated a population of some 370,000 crack users in the 27 Brazilian capital cities [37]. Most users have been shown to be young, socio-economically marginalized (e.g., poor/unemployed) and under-housed; further, most surveys typically only find a minority of crack users to be women [37–39].

In studies of different crack user populations attending in-patient or community-based treatment programs, only small minorities were females [40–42], offering only limited data on the distinct characteristics of female users, or potential differences between male and female users in Brazil. In a sample of pregnant crack users in in-patient detoxification treatment, 25% reported frequent daily crack use (>20 rocks per day); most had poly-substance use (e.g., tobacco, alcohol, cannabis). Close to half reported sex work, i.e. exchange of sex for money or drugs (45%) and crime involvement (e.g., robbery; 41%), and 15% were HIV-positive and 6% were HCV-positive [43]. Some other studies reported high prevalence of sex work and/or unsafe sex among female crack users in Brazil. In a sample of crack users in in-patient treatment, women were more likely to report sex-work involvement (and related HIV transmission risks) than men [44]. Community-based samples of crack users in Salvador and São Paulo reported high levels of unsafe sex and sex-for-money/drug-exchange activities as strategies to obtain crack among female participants, some of which also occurred in the distinct contexts of women obtaining crack for their intimate partners [35,45–50]. The pronounced sexual risk behavior patterns among female crack users in Brazil are directly linked to highly elevated transmission risk and prevalence of BBVs as well as exposure to violence and victimization [43,49,51]. Recent data suggest an increasing trend in the proportion of women crack users presenting in local health care facilities, which may either point to increasing use prevalence or increasing service utilization [52]. While substantive investments for substance use related health and treatment services (e.g., CAPS-AD, Therapeutic Community funding) have been made in Brazil, the need for special and targeted services for women has been emphasized given their distinct problem and needs profiles [35,43,44].

This paper examined gender-specific drug use, health, socio-economic and service use characteristics – and explored potential differences between males and females – among a community-recruited bi-site sample of young, regular crack users in Brazil.

## Methods

This study utilized data from a cross-sectional multi-site study of regular street-involved crack users recruited from impoverished neighborhoods in Rio de Janeiro (e.g., Jacarezinho) and Salvador (e.g., Pelourinho, Calabar, Ribeira, Fazenda Coutos and Valéria) with known extensive crack user populations (see [39] for details). Recruitment was facilitated by community-based contact persons (e.g., community workers) with direct access to the target population who disseminated basic study information to potential participants; study candidates were then assessed for eligibility by study field staff based on a brief screening protocol.

Eligibility criteria included: 1) crack use on three days + per week in the last three months; 2) 18–24 years of age; and 3) consent to participate in the full study protocol. Individuals experiencing acute intoxication or mental health problems, or displaying problematic behavior impeding assessment were not included. If eligible, and following written consent, study participants were assessed in a private study space at one of the community-based local study offices in either site.

A total of 175 (95 in Rio and 80 in Salvador) individuals were screened for eligibility; 15 were excluded for age or drug use criteria; a total of n = 160 study assessments (81/79) were completed between November 2010 and June 2011. Assessments consisted of an interviewer-administered questionnaire with 31 mainly quantitative items on socio-demographic, health and drug use characteristics, and service use/needs; in addition, blood specimens were collected by venipuncture and subsequently tested for HBAg, anti-HBc total, anti-HBs, total anti-HAV, anti-HCV, and anti-HIV using commercial enzyme immunoassays. All data and samples were sent to the Oswaldo Cruz Foundation (FIOCRUZ, RJ) for processing and analyses. Questionnaire data were scanned using Teleform® procedures and manually quality-checked; statistical analysis were conducted using STATA v.9.

One subject was excluded from analysis due to unspecified sex, resulting in a basic analysis sample of n = 159 (n = 124 males and n = 35 females). Descriptive analysis – e.g., proportions for categorical variables, mean/median values for continuous variables – for key variables of interest were computed and reported by sex. Statistical tests for differences between males and females (e.g., chi-squares for categorical variables, t-tests for continuous variables with p-value set at p < 0.05) were conducted.

Subsequently, a CHAID (‘Chi-squared automatic interaction detector’) algorithm was used to examine potential factors independently differentiating between males and female study participants [53,54]. The CHAID analysis essentially constitutes a non-binary tree classification method focusing on independent predictor factors or ‘nodes’ of classification into a dependent characteristic (here: sex). This occurs by a stepwise procedure in which the most significant variable (the largest χ2) is used to partition the sample into mutually exclusive subgroups. The cases in the emerging subgroups are further partitioned by the next most significant variable related to the dependent variable of interest, until there are no more significant variables. All variables were considered statistically significant at p-value <0.05. Cases with missing data for any of the variables examined (n = 6) were excluded from the CHAID analysis, resulting in an analysis sample of n = 153. The software used for this analysis was SPSS v.19.

Considering the dichotomous dependent variable of sex (male vs. female), the independent variables selected were: Education (some elementary school or less vs. completed elementary school or higher); housing status (stable vs. unstable or homeless); formal or informal work for income (yes vs. no); illegal activities for income (yes vs. no); begging/panhandling for income (yes vs. no); sex work for income and/or source to obtain drugs (yes vs. no); alcohol use (yes vs. no); marijuana use (yes vs. no); cocaine use (yes vs. no); daily crack use (yes vs. no); overdose (yes vs. no); sharing crack paraphernalia (yes vs. no); unprotected sex (yes vs. no); HIV (serology) status (positive vs. negative); Hepatitis A status (reagent vs. non-reagent); Hepatitis B status (reagent vs. non-reagent); Hepatitis C status (reagent vs. non-reagent); physical health status (‘good’ or better vs. ‘fair’ or worse); mental health status (‘good’ or better vs. ‘fair’ or worse); arrest (yes vs. no). Except for 'education' and ‘arrest’ (in past year), the reference period for all variables was ‘in past 30 days’ prior to the assessment.

The study protocol was approved by the Ethical Review Committee, Institute of Psychiatry, Federal University of Rio de Janeiro as well as the Brazilian National Ethics Committee (CONEP 519/2010).

## Results

Mean age of the samples was 21 years (range 18–24; Standard Deviation [SD] + 2.2) for females, and 22 years (18–24; SD 2.1) for males. Most respondents were of non-white (i.e., black or mixed) race, single and had some elementary school or less education. Significantly more females than males were characterized by unstable housing or homelessness. Close to half of men, and one third of females had been arrested in the past year. Both men and women had a variety of income sources; while the most common income sources for men were employment or some sort of paid work, more women relied on sex work and/or begging/panhandling for income generation, with illegal activities and support or transfer payments less common for both sexes (Table 1).

**Table 1:** **Socio**-**demographic characteristics**, **employment status**, **income generation and arrest history of sample**, **by sex**

	**Men** **(n = ** **124)** **mean** **(SD)**	**Women** **(n = ** **35)** **mean** **(SD)**	**Test statistic** ***t***	**P-** **value**
**Age**	20.7(2.1)	21.6(2.2)	2.174*	
	**N** (%)	**N** (%)	**χ** ^**2**^	
**Race**			0.3905	0.532
White	11 (9.0)	2 (5.7)		
Non-White/Other	111 (91.0)	33 (94.3)		
**Marital Status**			0.6712	0.413
Single/separ/div/widow	100 (80.6)	26 (74.3)		
Married or cohabitating	24 (19.4)	9 (25.7)		
**Education** ^**a**^			0.1736	0.677
Some elementary school or less	102 (82.3)	29 (85.3)		
Completed elementary school or higher	22 (17.7)	5 (14.7)		
**Housing status** ^**a**^ **[** 30 **]**			13.2492*	0.000
Stable housing	71 (57.7)	8 (22.9)		
Unstable housing or homeless	52 (42.3)	27 (77.1)		
**Income sources** **[** 30 **]**				
Formal or informal transfers (e.g., social assistance, money from family/friends)	35 (28.2)	10 (28.6)	0.0016	0.968
Paid employment/work	82 (66.1)	6 (17.1)	26.503***	0.000
Illegal activities (including drug-related work)	25 (20.2)	4 (11.4)	1.396	0.237
Sex work	3 (2.4)	16 (45.7)	48.627***	0.000
Begging/panhandling	19 (15.3)	14 (40.0)	10.107***	0.001
**Arrest history**				
Detained by police (past year)	56 (45.2)	11 (31.4)	2.111	0.146

*p < 0.05, **p < 0.01, ***p < 0.001.

^a^Subjects with missing data not included. [30]: in the last 30 days.

Participants had an average history of 4 (SD: 3.0; males) and 5 (SD: 3.4; females) years of crack use, and used an average of 10 (SD: 11) and 8 (SD: 6) rocks of crack per day, respectively. The majority of both males and females were current tobacco users; while men also commonly reported current use of marijuana, alcohol or cocaine, women reported lower use prevalence of these drugs. The use of other drugs was either marginally small or non-existent (Table 2).

**Table 2:** **Crack and other drug use characteristics of sample**, **by sex**

	**Men** **(n** ** = 124)** **M** **(SD)**	**Women** **(n = ** **35)** **M** **(SD)**	**Test statistic t**	**P-** **value**
Number of years of crack use	4.0 (3.0)	4.9 (3.4)	1.547	
Number of crack rocks used per typical day	10.1 (10.5)	8.3 (5.9)	0.925	
	**N** (%)	**N** (%)	**χ** ^**2**^	
Daily crack use [30]	66 (53.2)	24 (68.6)	2.617	0.106
**Main modes of crack use**				
Smoking mixed crack and tobacco	7 (5.7)	2(5.7)	0.0002	0.988
Smoking mixed crack and marijuana	29 (23.4)	1 (2.9)	7.5154*	0.006
Smoking crack using a can	8 (6.5)	2 (5.7)	0.0252	0.874
Smoking crack using a plastic cup	50(40.3)	22 (62.9)	5.5941*	0.018
Smoking crack using a pipe	28(22.6)	8 (22.9)	0.00123	0.972
Shared crack smoking implements [30]^a^	72 (62.1)	21 (60.0)	0.0487	0.825
**Other drug use** **[** 30 **]** ^**a**^				
Alcohol	68 (55.7)	9 (25.7)	9.8100**	0.002
Tobacco	97 (78.2)	31 (88.6)	1.8614	0.172
Marijuana	88 (71.5)	18 (51.4)	4.9936*	0.025
Amphetamines or LSD	0 (0.0)	0 (0.0)	-	-
Cocaine	55 (45.8)	4 (11.4)	13.6049***	0.000
Benzodiazepines	3 (2.4)	0 (0.0)	0.8702	0.351
Opioids	0 (0.0)	0 (0.0)	-	-
Inhalants/Solvents	4 (3.4)	2 (5.9)	0.4325	0.511

*p < 0.05, **p < 0.01, ***p < 0.001.

^a^subjects with missing data not included. [30]: in the last 30 days.

Very few participants reported a lifetime injection drug history, or a recent overdose experience. While majorities of both males and females reported unsafe sex activities, more females had recent sex-for-drug/money exchanges. More women had been tested for HIV and HCV; almost five times as many women as men were determined to be HIV+. Close to half of both men and women rated their physical health as ‘good’ or better. Similar rates reported physical health problems; most of those with problems did not receive medical attention yet would have liked to do so. Similar patterns emerged for mental health, except that more men than women rated their mental health as ‘good’ or better. Twice as many women recently utilized any kind of social, health or treatment service; social services were most commonly used, followed by health and treatment services, respectively. Substantive majorities among both sub-groups indicated that they would use specific help services for drug users if available to them (Table 3).

**Table 3:** **Health risk**, **status and service utilization characteristics of sample**, **by sex**

	**Men** **(n** ** = 124)** **N (%)**	**Women** **(n = ** **35)** **N (%)**	**Test statistic χ** ^**2**^	**p-** **value**
**Sexual Risks**				
Had sex without a condom [30]	75 (60.5)	22 (62.9)	0.0646	0.799
Had sex in exchange for drugs in [30]	5 (4.0)	10 (28.6)	19.2377***	0.000
**Health status**				
Drug overdose [30]	8 (6.5)	1 (2.9)	0.6257	0.429
Injection drug use (lifetime)	2 (1.6)	0 (0.0)	0.5717	0.450
Oral sores/injuries [30]	18 (14.8)	5 (14.3)	0.0048	0.945
Ever tested for HIV	27 (22.3)	26 (76.5)	34.5957***	0.000
HIV + (serology)	5 (4.1)	6 (18.9)	7.8139**	0.005
Ever tested for Hep C	9 (7.6)	18 (60.0)	43.9874***	0.000
Hep C + (HCVAB serology)	0 (−)	1 (3.0)	3.7210	0.054
Hep B serology	17 (13.9)	9 (28.1)	3.6379	0.056
Hep A serology	99 (81.2)	29 (90.6)	1.6227	0.203
**Physical health problems [** 30 **]**	52 (42.6)	16 (45.7)	1.0754	0.584
Received medical attention^b^	7 (12.7)	5 (31.3)	3.0277	0.082
Would like to receive medical attention^b^	40 (78.4)	12 (75.0)	3.1357	0.077
**Self**-**rated physical health** ^**a**^			0.5902	0.422
‘Good’ or better	49 (41.5)	12 (34.3)		
**Mental health problems [** 30 **]**	58 (46.8)	15 (42.9)	0.8137	0.666
Received medical attention^b^	1 (1.75)	0 (−)	0.2491	0.618
Would like to receive medical attention^b^	38 (66.7)	10 (71.4)	0.1164	0.733
**Self**-**rated mental health**			4.4885*	0.034
‘Good’ or better	63 (52.9)	11 (32.4)		
**Service utilization**				
Accessed social, health or drug treatment services [30]	37 (29.8)	20 (57.1)	8.8485**	0.003
**Type of service** **[** 30 **]** ^**c**^				
Social services	24 (19.5)	16 (45.7)	9.8937**	0.002
Health services	12 (10.4)	8 (22.9)	4.2299*	0.040
Drug treatment service used [30]	3 (3.2)	2 (6.5)	0.6654	0.415
Would use service for DU’s, if available.	98 (79.0)	27 (77.1)	0.2578	0.879

*p < 0.05, **p < 0.01, ***p < 0.001.

^a^missing values.

^b^of those who reported problems.

^C^includes multiple responses. [30]: in the last 30 days

**Figure 1:**
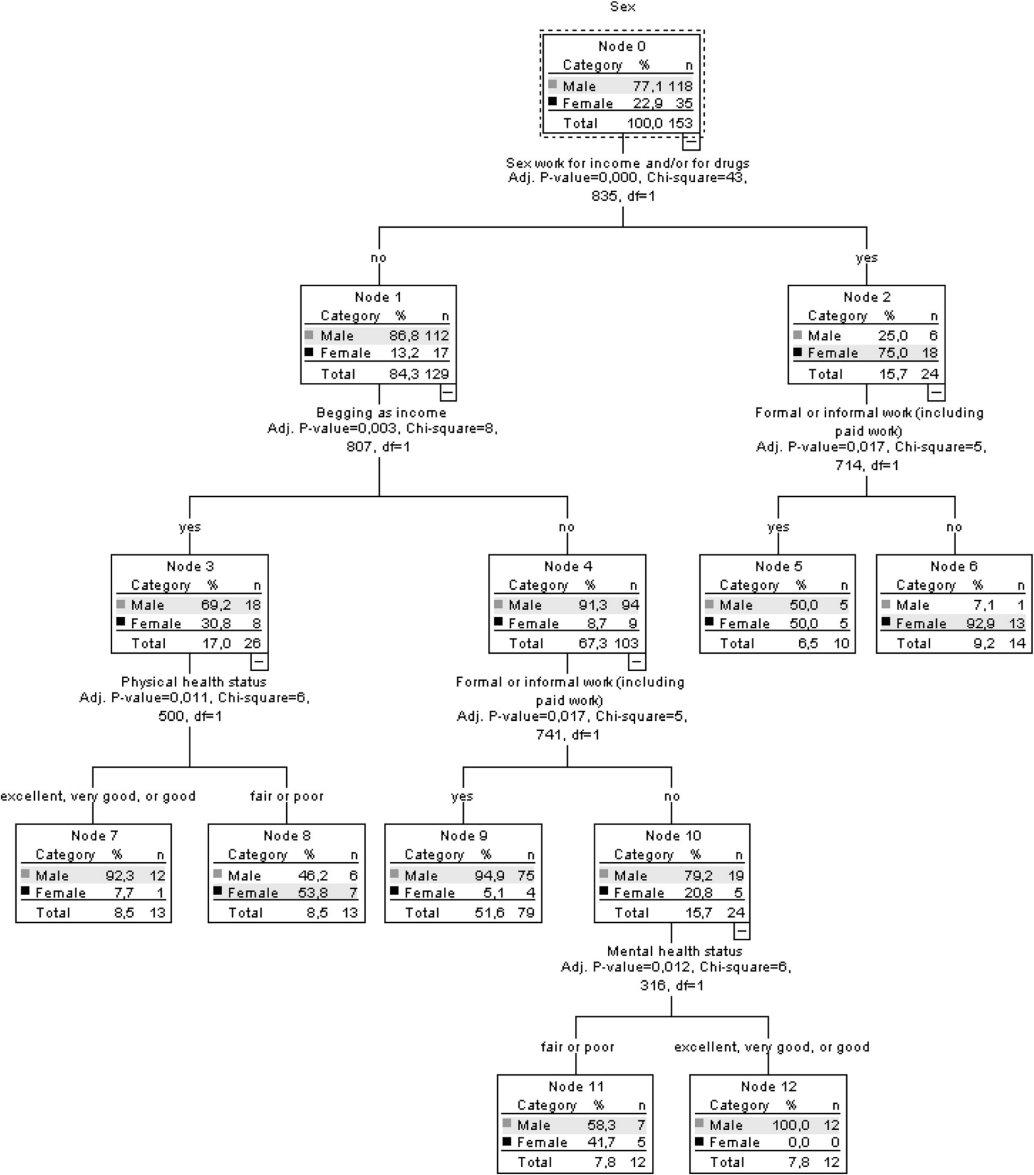
**Results of chi-squared automatic interaction detector (CHAID) model for study sample with ‘sex’ as dependent outcome variable.**

In the results of the CHAID analysis (Figure 1), the most important variable differentiating male and female participants was sex work for income and/or to get drugs (p < 0.000). Among the individuals involved in sex work, male respondents more frequently than females reported involvement in formal/informal work (p = 0.017). Among participants without sex work involvement, engaging in begging/panhandling for income (p = 0.003) emerged as the differentiating factor by sex. Among respondents with begging for income, physical health status (p = 0.011) was the main differentiating factor by sex; among respondents not involved in begging, formal or informal work (p = 0.017), again, was the principal differentiating factor; finally, among respondents who indicated neither begging nor formal/informal work as income sources, mental health status (p = 0.012) emerged as the main differentiating factor between males and females.

## Discussion

Our study’s results revealed several key areas of differences between males and females in our bi-city sample of young crack users in Brazil, with implications for interventions and further research.

First, our findings – both from univariate and the CHAID analyses – indicated key differences in income generation behaviors between male and female participants. While most men reported some form of paid work, the largest proportion of women reported involvement in sex work and/or begging/panhandling for income generation. These results extend findings from several other studies with crack users elsewhere, suggesting pronounced patterns of ‘gendering’ related to income sourcing in this population. That is, while men are more commonly involved in legal or illegal ‘work’ or crime activities, women commonly rely on commercial sex or other forms (e.g., begging/panhandling) of income generation [24,26,55,56]. These patterns bring several implications. First – while our data did not specify what ‘work’ concretely meant – the predominant exclusion of women from more ordinary ‘work’ activities and primary reliance on sex work or begging may entail an even more pronounced socio-economic ‘marginalization’ of female users than is already generally the case for this population [55,57,58]. These marginalization dynamics may further negatively influence the risks and consequences of drug use, as well as engagement with or access to key basic (e.g., social or health) services and interventions, and hence amplify the overall negative circumstances of women crack users. Second, our results confirm findings of other studies from different jurisdictions (including some from Brazil) documenting the common and intensive involvement of female crack users in sex work [43,51,59,60]. Sex work involvement among female crack users is well documented to be strongly associated with key health risks – e.g., unprotected sex, unsafe sex practices, multiple sex partners, acute BBV transmission, often occurring in the context of crack use ‘binges’ – as well as exposure to violence and related victimization [20,21,23,25,27,60]. Commonly, commercial sex work among female crack users occurs within the imbalanced power dynamics of intimate relations with male crack user partners, where women are charged with procuring funds to acquire drugs for themselves and their partner [18,49]. Given the high prevalence of sex work for income among female users and its key consequences (see also below) in our sample, implementing interventions to reduce sex work involvement and/or or related harms among female crack users in Brazil constitutes an urgent intervention need.

Second, our data indicate higher prevalence, and more diversified patterns of (current) poly-drug use (including alcohol, marijuana, and cocaine) among male than among female crack users. These differences are notable, in particular since – with the exception of cocaine co-use – they are mostly absent when ‘lifetime’ histories for these drugs (data not shown) are considered; moreover, almost 4 in 5 women (twice as many compared to men) reported a ‘lifetime’ history of inhalants/solvents. These gendered co-drug use patterns – and specifically the more pronounced current ‘mono’-crack use profiles among females – point to a number of potential dynamics. The drug use pathways into crack use may differ between males and females; further, it is a worthwhile question whether the disappearance of inhalant/solvent use among females is mainly an ecologically driven or a ‘drug use career’ effect [1,4]. While tobacco and marijuana are used by most female crack users (and have been shown in other crack user populations to be used in combination with crack in order to modify effects) [61,62], women may either not desire, or may not have available or be able to afford the other drugs – alcohol and/or cocaine – which were commonly co-used by men. Additional inquiries – e.g., including qualitative/ethnographic investigations – should investigate these differences, as these may entail implications for health consequences and intervention/treatment needs (e.g. in respect to poly-dependence, physical co-morbidities, etc.) [4,63,64].

A third key area of differences concerns health risk and status indicators. While men and women indicated overall similar physical health profiles, the differences in BBV status – in particular HIV, with women reporting elevated levels – are noteworthy. Since drug injecting histories – the most common cause of HIV transmissions among illicit drug users – are close to absent in our study population, we must assume that most HIV infections were caused by sexual transmission pathways [27,29,65]. On this basis, our data suggest that the common involvement in sex work activities and associated sexual risk behaviors among females in our sample constituted a primary risk factor for increased BBV transmission and status levels [35,45,51,59,66,67]. Studies conducted elsewhere have found crack use to be an independent predictor of HIV and other BBV (e.g., HCV) transmissions among drug user populations [68–71]. Our data underscore the urgent need for targeted HIV and other BBV prevention and treatment measures in the study population, yet primarily among female crack users, with a primary emphasis on sexual risk reductions. Examples of peer- or community-based interventions – e.g., outreach programs and/or brief cognitive-behavioral interventions – aiming at sex-related risk behaviors among female crack users have been implemented elsewhere with positive results [72–75].

A further difference is related to the substantially lower levels of self-rated mental health among female participants. While these differences were not shown for mental health ‘problems’, and while the self-assessment of one’s own mental health status constitutes a subjective exercise, somewhat higher levels of certain mental health problem symptoms and/or disorders among female drug – including stimulant – users have been documented elsewhere; in particular, depression, anxiety and PTSD symptom levels have been found to be higher among female users [1,2,9,76]. These mental health problems are commonly associated with stimulant misuse, and hence may burden female participants in our study population more distinctly than men and, consequently, lead to more intensive and/or problematic substance use behaviors [77–79]. While these data underscore the need for interventions and care focusing on co-occurring mental health problems among female crack users, such problems are common among male users as well and require general and integrated attention in targeted services for the study population.

Finally, we found substantively higher social and health service utilization rates – including higher HIV and HCV testing rates – among female crack users in our sample, [80]. While women in Brazil generally indicate higher health service attendance, multiple possible explanations and implications exist for these differences in our study population [81]. Overall, our data seem to suggest a somewhat – yet only partially closer – connection and involvement of female crack users with care services, which may suggest that women in our study’s local contexts find these services more accessible or useful for their needs (even though most services were utilized by less than half of women, meaning that the majority of women did not access any of these services). Previous data has documented that – despite substantive recent investments in and expansions of psycho-social and other treatment services for psychoactive drug users in Brazil (e.g., CAPS-AD; [82]) – many crack users experience major barriers and problems with existent service offers, and only few utilize or access them [33,83,84]. The observed sex-based differences in service utilization may have other gender-specific reasons, e.g., higher use of universal pre-natal care related services – including sexual health and BBV testing – offered to women in Brazil [51,85,86]. While the present study did not collect relevant data to explain these dynamics in more detail, it is documented that many female crack users in Brazil have children, and thus pre-natal histories [35,37,43]. Female crack users may also be better connected to basic (e.g., outreach) care through local services or interventions specifically targeting sex workers [50,87,88].

Potential implications arise from these differential service utilization profiles. Especially given the predominant general ‘marginalization’ of crack users, improved connections or involvement with social or health services would likely allow for improved delivery of or referrals to other – including general health, prevention or treatment – services; this is much more difficult with drug users who are completely disconnected from the service system [57,89,90]. It is further documented that BBV (e.g., HIV, HCV) testing is important for both creating awareness in regards to disease status and risk behavior changes, as well as for initiating effective treatment among risk populations [91–95]. Thus, the observed differential service utilization patterns between male and female crack users ought to be better understood towards improved intervention development and delivery.

Notably, the service utilization differences did not hold for treatment utilization specifically, where less than one in ten participants in both groups reported any recent involvement. This may imply various reasons, including lack of perceived need for treatment, limited availability or access barriers; recent data have suggested that all of these factors may be at work in the study population [33]. While other studies have emphasized distinct treatment and service needs for women drug users, it appears – despite recent expansions of addiction treatment services in Brazil – that major gaps in treatment offers, access and utilization for crack users in Brazil in general continue to exist which urgently ought to be addressed [13,33,35,40,44,83]. This observation is supported by the high proportion of (both male and female) users expressing desire to utilize improved service offers for drug users if these were available.

Our study has several potential limitations. It relied on a community-based convenience sample which may include selection biases and compromise generalizability of results; in addition, most data (except for biological tests) were based on self-report which are not objectively verifiable; however our study included key methodological provisions (e.g., subject anonymity, study procedures independent from service provision, etc.) for enhanced validity, and data from similar study designs have shown to be valid [96]. The sample, and key data values reported, were relatively small, potentially compromising analytical power. The study protocol did not include clinical or diagnostic assessments (e.g., for dependence) to formally assess care (e.g., treatment) needs. The gender sub-samples were comprised of participants from two different study locations, where ecological or contextual differences (e.g., drug availability or costs, use cultures, service offers, etc.) may have influenced individual behaviors or characteristics.

## Conclusions

Our study documented several important differences in drug use, health and service indicators between young male and female crack users in Brazil. Given the prevalence of crack use in Brazil, these data illustrate the urgent need for gender-specific interventions in several (including targeted prevention, treatment) realms towards reducing the extensive related health and social problem burden of crack use.

## Abbreviations

Anti-HAV: Hepatitis A virus antibody
Anti-HBc: Hepatitis B core antibody
Anti-HBs: Hepatitis B surface antibody
Anti-HCV: Hepatitis C virus antibody
BBV: Blood borne virus
CAPS-AD: Centro de Atencao Psicossocial Alcool e Drogas
CHAID: Chi-squared automatic interaction detector
FIOCRUZ: Fundação Oswaldo Cruz
HBAg: Hepatitis B antigen
HCV: Hepatitis C virus
HIV: Human immunodeficiency virus
PTSD: Post-traumatic stress disorder
SD: Standard deviation
SPSS: Statistical package for the social sciences
STD: Sexually transmitted disease
